# Spatial Patterns and Multilevel Analysis of Factors Associated with Antenatal Care Visits in Nigeria: Insight from the 2018 Nigeria Demographic Health Survey

**DOI:** 10.3390/healthcare9101389

**Published:** 2021-10-18

**Authors:** Obasanjo Afolabi Bolarinwa, Barbara Sakyi, Bright Opoku Ahinkorah, Kobi V. Ajayi, Abdul-Aziz Seidu, John Elvis Hagan, Zemenu Tadesse Tessema

**Affiliations:** 1 Department of Public Health Medicine, School of Nursing and Public Health, University of KwaZulu-Natal, Durban 4091, South Africa; bolarinwaobasanjo@gmail.com; 2 Department of Population of Health, University of Cape Coast, Cape Coast PMB, Ghana; barbara.sakyi@stu.ucc.edu.gh (B.S.); abdul-aziz.seidu@stu.ucc.edu.gh (A.-A.S.); 3 School of Public Health, University of Technology Sydney, Sydney, NSW 2007, Australia; brightahinkorah@gmail.com; 4 Department of Health and Kinesiology, Texas A&M University, College Station, TX 77843, USA; kobiajayi@gmail.com; 5 College of Public Health, Medical and Veterinary Sciences, James Cook University, Townsville, QLD 4811, Australia; 6 Department of Health, Physical Education, and Recreation, University of Cape Coast, Cape Coast PMB, Ghana; 7 Neurocognition and Action-Biomechanics-Research Group, Faculty of Psychology and Sport, Sciences, Bielefeld University, Postfach 10 01 31, 33501 Bielefeld, Germany; 8 Department of Epidemiology and Biostatistics, Institute of Public Health, College of Medicine and Health Sciences, University of Gondar, Gondar P.O. Box 196, Ethiopia; zemenut1979@gmail.com

**Keywords:** antenatal care visits, multi-level analysis, Nigeria, pregnant women, spatial analysis

## Abstract

Despite global progress towards antenatal care (ANC) uptake, ANC utilization in a number of countries in sub-Saharan Africa, such as Nigeria, is low. Although several studies have identified the determinants and factors associated with ANC services utilization in Nigeria, there is a gap in knowledge about the spatial patterns in ANC use. Therefore, this study aims to map the spatial distribution and factors associated with ANC visits in Nigeria. A cross-sectional dataset was obtained from the 2018 Nigeria Demographic and Health Survey. A total of 20,003 women aged 15–49 were considered in this study. Both spatial and multilevel analyses were carried out. The results were presented in spatial maps and adjusted odds ratios (aOR) at a 95% confidence interval (CI). Hot spot areas (high proportion of an incomplete ANC visit) were located in Sokoto, Kebbi, Zamfara, Katsina, Kano, Jigawa, Bauchi, Niger, Borno, Gombe, and Bayelsa. Regional disparities in incomplete ANC visits were found in this study. Maternal age, maternal education, partner’s level of education, working status, ethnicity, parity, religion, exposure to media, place of residence, wealth index, region, and community literacy level were factors associated with incomplete ANC. There is a need to consider these factors in the design and strengthening of existing interventions (e.g., mini-clinics) aimed at increasing ANC visits to help attain maternal health-related Sustainable Development Goals by 2030. The regional disparities in incomplete ANC visits also need to be considered by encouraging pregnant women in hotspot areas to attend ANC visits.

## 1. Introduction

Antenatal care (ANC) is an essential element in the continuum of reproductive health care for preventing preventable pregnancy-related morbidity and mortality. It provides a platform for integrated health functions, including health promotion, screening and diagnosis, and disease prevention before and after pregnancy [1]. ANC also improves newborn health outcomes by reducing stillbirths and other neonatal deaths [2]. Most maternal and infant deaths can be prevented or treated when women receive ANC [1,2]. Unfortunately, in 2017 alone, 297,000 women died from preventable pregnancy and childbirth-related causes, with 86% of these deaths occurring in low-and-middle-income countries, according to the World Health Organization [3]. However, sub-Saharan Africa (SSA) alone accounts for up to two-thirds or 196,000 of global maternal deaths [3]. 

Despite global progress towards ANC uptake, ANC utilization in SSA is low, just as in Nigeria. Globally, 71% of women utilize ANC; approximately 95% of women access ANC in developed nations compared to 69% in SSA who manage to attend at least one ANC visit [2,4,5]. In Nigeria, reports indicate that the proportion of women who do not attend ANC services is between 33.9% and 34.9%. At the same time, only 51.0% receive four or more ANC visits during their pregnancy, according to Fagbamigbe and Idemudia [4]. The poor ANC utilization in Nigeria could be attributed to the unacceptably high rates of pregnancy-related deaths, earning Nigerian women a 1 in 22-lifetime risk of dying during pregnancy, childbirth, or the postpartum period [6,7]. Approximately 20% of all global maternal deaths occur in Nigeria, and between 2005 and 2015, roughly 600,000 maternal deaths and over 900,000 maternal near-misses were recorded, according to the World Health Organization [6]. Although there is progress in ANC utilization and reduced maternal deaths in the country, Nigeria still performs poorly compared to neighboring countries in Africa, according to Fagbamigbe and Idemudia [4,8]. Moreover, the maternal mortality ratio of 800 per 100,000 live births in Nigeria continues to be clearly above the African and global average of 500 and 210, respectively [6,8]. Suboptimal ANC utilization in Nigeria is a serious threat to the actualization of the Sustainable Development Goals (SDGs) by 2030.

Studies have demonstrated that sociodemographic factors such as age, wealth index, geographical location, educational status, husband occupation, marital status, and socioeconomic status are significantly associated with ANC utilization [2,4,5,9,10,11,12]. For instance, a cross-sectional study conducted in Nigeria by Fagbamigbe and Idemudia [4] found a five-fold increase in ANC utilization among women in the wealthiest quintile. Another study using the 2013 Nigeria Demographic and Health Survey (NDHS) data assessed ANC utilization using concentration curves and indices. This study found that the likelihood of not receiving ANC was highly concentrated among poor women living in northern Nigeria [10]. Other factors such as inadequate financing, the availability of competent and skilled health care providers—particularly in rural areas—distance to ANC facilities, and inadequate or poor-quality health services are also associated with ANC use in Nigeria and elsewhere [13,14]. Additionally, other factors related to poor ANC use include misaligned communication among formal and informal health care providers and unprofessional conduct such as disrespect of patients’ privacy, confidentiality, and traditional beliefs [7,15]. 

However, the poor health outcomes and under-utilization of ANC in Nigeria do not occur in isolation, rather they are a result of multifactorial issues within the health care sector in the country. For example, the number of health care providers in the country is painfully low, with about 0.381 physicians per 1000 people. Simultaneously, there is a lack of hospital beds, with only 0.5 hospital beds per 1000 people [16]. Furthermore, delivery and access to care are impeded by suboptimal or lack of technological health care tools. The literature suggests that Nigeria has poor health record management practices, which is mostly due to long-time neglect, inadequate funding, long waiting times at health facilities, and poor storage and retrieval of patients’ health records [17]. Indeed, these issues affect health care utilization and subsequently, ANC uptake. 

Although several studies have identified the determinants of ANC utilization in Nigeria, there is a gap in knowledge about the spatial patterns connected to ANC use. The spatial analysis provides a robust understanding of population factors and their association with health service environment on ANC use. This also helps to solve complex location-oriented problems and give a clear and better understanding of where and what is occurring in the study area. It helps with understanding the characteristics of places and the relationships between those places. Spatial analysis lends new perspectives to decision-making [11]. Since Nigeria still lags behind in achieving the recommended World Health Organization ANC visits, investigating the multi-level factors and spatial distribution of ANC use in Nigeria is an important step to closing existing gaps. Therefore, this study aims to map the spatial distribution and factors associated with ANC visits in Nigeria.

## 2. Methods and Materials 

### 2.1. Data Source 

Data were obtained from the 2018 Nigeria Demographic and Health Survey (NDHS). The NDHS is a cross-sectional survey that gathers data on health information on men, women, and children. The NDHS is nationally representative and collects data from 36 administrative units and the Federal Capital Territory using a two-stage sampling process. The survey data cover various health topics, including antenatal care visits [18]. The NDHS methodology is reported in the National Population Commission (NPC) and International Center for Migration (ICF). We followed the guidelines for enhancing the reporting of observational studies in epidemiology when producing this publication [19]. The dataset can be downloaded from https://dhsprogram.com/data/available-datasets.cfm (accessed on 20 March 2021). 

### 2.2. Sample Size Determination and Inclusion Criteria

The primary sampling unit for the survey consisted of samples chosen at random from clusters. A total of 20,003 women aged 15–49 were considered for this study, based on data from the children’s recode file. The selection process involved the selection of eligible households from the clusters. All eligible interviewed women of reproductive age between 15 and 45 years were initially considered; however, after the exclusion of women without a complete information on ANC visits and other variables of interest, the initial sample selection then dropped from 34,082 to 20,003 respondents, and this is the sample size included in the study analysis. 

### 2.3. Outcome Variable

The outcome variable in this study was the “number of ANC visits.” In this study, the number of ANC visits was defined as the percentage of women aged 15–49 who had a live birth in a given time period that received ANC services during pregnancy. This variable was derived from the question, “How many times did you receive ANC during this pregnancy?” The responses ranged from 0 to 20. Following the revised World Health Organization guidelines on the recommended number of ANC visits [8], the responses were categorized as <8 visits or ≥8 visits, where ≥8 visits were considered complete ANC visits and <8 incomplete ANC visits. Similar categorizations have been used in previous studies [20,21,22]. 

### 2.4. Independent Variables 

Based on theoretical and practical significance and the availability of the variables in the dataset, we considered both individual- and household-level factors in our study. The selection of the variables was influenced by their association with the number of ANC visits in previous studies [20,21,22]. 

### 2.5. Individual-Level Factors

The individual-level factors were maternal current age (15–24, 25–34, 35 and above), maternal level of education (no education, primary, secondary and above), partner’s level of education (no education, primary, secondary and above), marital status (currently married, cohabitating), working status (working vs. not working), ethnicity (Hausa, Yoruba, Igbo, others), parity (1, 2, 3, 4 and above), distance to health facility (big problem, not a big problem), religion (Christianity, Islam, traditionalist and others), and exposure to media (no, yes). 

### 2.6. Household-Level Factors

The household-level factors were place of residence (urban vs. rural), wealth index (categorized by DHS as poorest, poorer, middle, richer, and richest, and the same categories were used in this study), region (North Central, North East, North West, South East, South-South, South West), sex of household head (male, female), community literacy level (low, medium, high), and community socioeconomic status (low, medium, high). 

### 2.7. Statistical Analyses

Both spatial and multi-level analyses were carried out. 

#### 2.7.1. Multi-Level Analysis 

A two-level multi-level binary logistic regression was built to assess the individual- and household-level factors associated with incomplete ANC visits in Nigeria. Women were nested within households in the modeling, and subsequently, households were nested within clusters. Clusters were considered as random effects to account for the unexplained variability at the household level. A total of four models were fitted. Firstly, we fitted an empty model, model 0, which contained no predictors (random intercept). After that, model I contained individual-level variables alone, model II included household-level variables and model III was the complete model that comprised both individual-level and household-level variables. The odds ratio and its corresponding 95% confidence intervals (CIs) were provided for models I–III. These models were fitted by a Stata command “melogit.” The log-likelihood ratio (LLR), Akaike Information Criterion (AIC) measure, and Schwarz’s Bayesian Information Criterion (BIC) were used for model comparison. The best fit model has the highest log-likelihood and the lowest AIC [23]. We also tested for multicollinearity by using variance inflation factor (VIF), which showed no collinearity among the independent variables (Mean VIF= 1.79, Maximum VIF= 2.99, and Minimum VIF= 1.07). In the individual populations, sample weight (v005/1,000,000) was used in all analyses to account for over-and under-sampling, whereas the “svy” command was used to account for the survey’s complex nature, which also helps in the generalizability of the findings. Stata version 16.0 (Stata Corporation, College Station, TX, USA) was used for statistical analyses.

#### 2.7.2. Spatial Analysis 

##### Spatial Autocorrelation 

Spatial autocorrelation analysis was performed to check whether there is a clustering effect on incomplete ANC visits in Nigeria. This analysis result gives Global Moran’s I value, Z-score, and *p*-value for deciding whether the data are dispersed or random or clustered. A Moran’s I value close to positive 1 indicates a clustering effect, close to negative one indicates dispersed, and close to zero random. If the *p*-value is significant and the I value is close to zero, this means that incomplete ANC visit had a clustering effect. 

##### Hot Spot Analysis (Getis-OrdGi* Statistic)

The hot spot analysis tool gives Getis-Ord or Gi* statistics for a cluster in the dataset. Statistical values such as Z-score and *p*-value are computed to determine the statistical significance of clusters. Results of the analysis with a high GI* value mean hot spot areas (high prevalence of an incomplete ANC visit), and a low GI* value means cold spot areas (low prevalence of an incomplete ANC visit).

##### Spatial Interpolation or Prediction 

Spatial prediction is one of the techniques of estimating unsampled areas based on sampled areas. In the 2018 NDHS, a total of 1400 clusters were selected to take a sample for this area that is believed to be representative of the country. An Ordinary Kriging prediction method was used for this study to predict an incomplete ANC visit in unobserved areas of Nigeria.

##### Spatial Scan Statistical Analysis

Bernoulli’s purely spatial model was applied to identify primary and secondary, and incomplete ANC visit clusters using 1393 enumeration areas. SatTscan software (Havard Medical School, Boston, MA, USA) was used for the analysis. First, the dataset was managed as appropriate for SaTScan software. Women who had no complete ANC visits were taken as cases, and women who had complete ANC visits were taken as controls. The cluster number, longitude, and latitude data were obtained from the GPS dataset. The cluster size of less than 50% of the population was taken as the upper bound. A 999 Monte Carlo replication was used for this study. Based on the above criteria, primary and secondary clusters were identified.

## 3. Results

### 3.1. Sociodemographic Characteristics of Respondents 

A total of 20,003 women of reproductive age were included in the study. At the individual level, 9665 (48.32%) of the respondents were aged 25 to 34. A total of 9245 (46.22%) had no education, while 9886 (49.42%) of the respondents’ partners had secondary education and above. A total of 19,387 (96.92%) of the respondents were currently married, and 12,326 (61.62%) of the women had mass media exposure. At the household level, 12,266 (61.32%) of the study women resided in rural areas, 4464 (22.32%) were from the poorest wealth index households, 7269 (36.34%) were residing in the North West region, 6786 (33.93%) were from a community with a high literacy level, and 11,957 (59.77%) were from a community with low socioeconomic status. All the individual and household-level factors showed significant associations with ANC visits in Nigeria (Table 1).

### 3.2. Spatial Autocorrelation 

**Figure 1:**
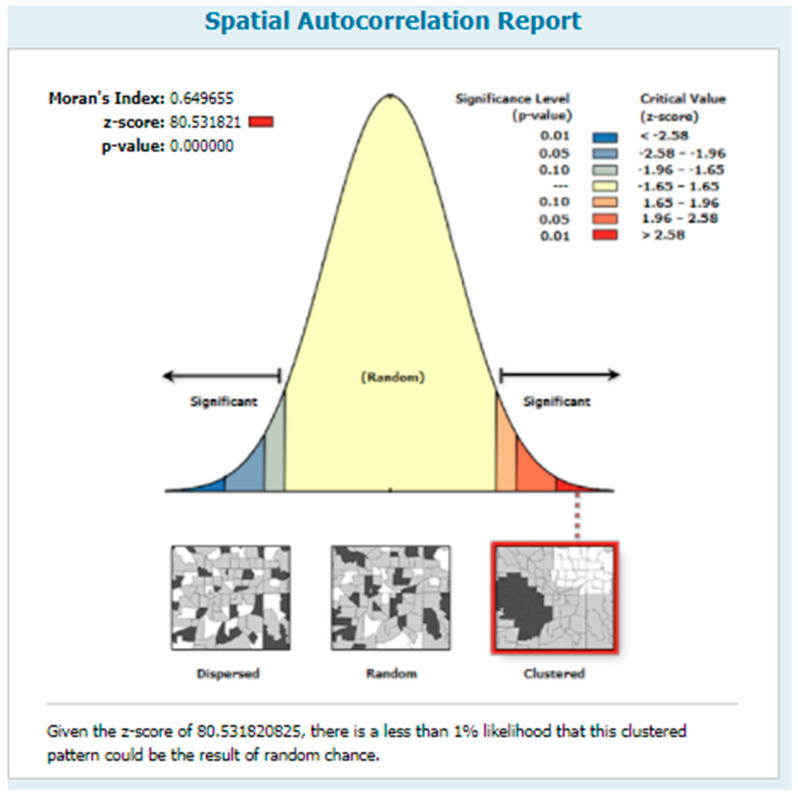
Spatial autocorrelation of incomplete ANC visits in Nigeria, 2018.

Spatial autocorrelation analysis was carried out to check whether an incomplete ANC visit was random or not. According to the Global Moran’s I value z-score and *p*-value of *p* < 0.0001, an incomplete ANC visit was clustered in Nigeria across all regions (Figure 1).

### 3.3. Hot Spot Analysis 

**Figure 2:**
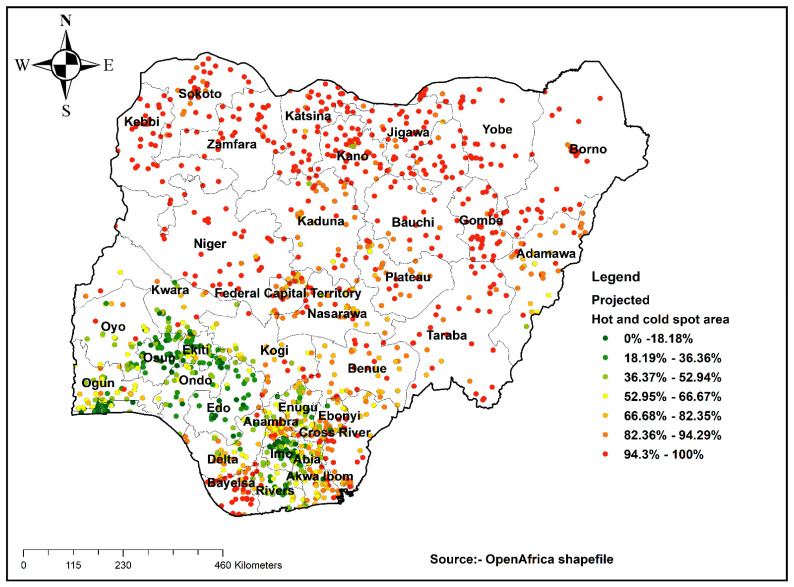
Hot spot analysis result of incomplete ANC visits in Nigeria, 2018.

Hot spot analysis was conducted using Getis-Ord GI* analysis to detect hot and cold spot areas. Hot spot areas (high proportion of an incomplete ANC visit) were located in Sokoto, Kebbi, Zamfara, Katsina, Kano, Jigawa, Bauchi, Niger, Borno, Gombe, and Bayelsa. The cold spot areas were located in Lagos, Osun, Ekiti, Kogi, Enugu, Imo, Abia, River, and Oyo (Figure 2). 

### 3.4. Prediction of an Incomplete ANC Visit

**Figure 3:**
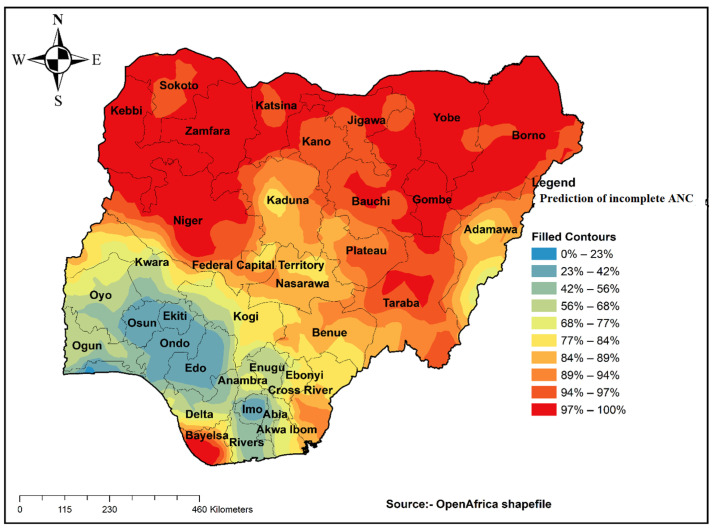
Interpolation of incomplete ANC visits in Nigeria, 2018.

Prediction analysis was performed. The result gives the prevalence of an incomplete ANC visit for the unsampled area of Nigeria based on the sample area. The prediction revealed that areas such as those shown in red are high-risk areas for an incomplete ANC visit in Nigeria (Figure 3).

### 3.5. Spatial SaTScan Analysis of Stillbirth Bernoulli-Based Model

**Figure 4:**
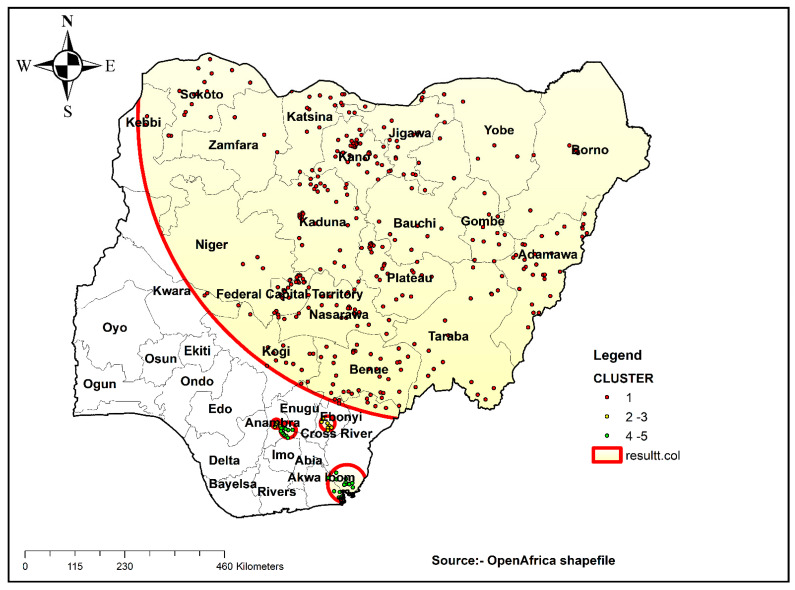
SaTScan analysis result of incomplete ANC visits in Nigeria, 2018.

Most likely (primary) and secondary clusters of an incomplete ANC visit were identified. A total of 336 clusters were identified: 336 were primary, and 8 were secondary clusters. The primary clusters’ spatial window was located in the Northern part of Nigeria (12.458115 N, 10.398881 E)/690.22 km, and log-likelihood ratio (LLR) of 1307 and relative risk (RR) 1.79, at *p* < 0.0001. It showed that women within the spatial window had 1.79 times higher risk of an incomplete ANC visit than women outside the window. Likewise, the secondary clusters were centered at (6.224563 N, 7.971626 E)/16.54 km radius, LLR of 11.90, and relative risk (RR) 1.28 at *p*-value 0.0062. This showed that women within the spatial window had 1.28 times higher risk of an incomplete ANC visit than children outside the window (Table 2, Figure 4).

### 3.6. Multi-Level Fixed Effects (Measures of Associations) Results

The factors associated with an incomplete ANC visit in Nigeria at the individual level include maternal age, maternal education, partner’s level of education, working status, ethnicity, parity, religion, and exposure to media, while the place of residence, wealth index, region, and community literacy level were factors associated with incomplete ANC at the household level (Table 3). 

### 3.7. Random Effects (Measures of Variations) Results

The empty model (model 0), as shown below in Table 3, depicted a substantial variation in the likelihood of incomplete ANC visits in Nigeria across the Primary Sampling Unit (PSU) clustering (σ2 = 5.33; 95% (CI = 4.73–6.02)). Model 0 indicated that 62% of the variation in incomplete ANC visits in Nigeria was attributed to Intra-Class Correlation variation, i.e., (ICC = 0.62). The between-cluster variation decreased to 29% (0.29) in model I (individual-level variables only). In the household-level variables (model II), the ICC decreased to 26% (ICC = 0.26). In comparison, the ICC declined further to 24% (ICC = 0.24) in the complete model with both the individual and household-level factors (model III). This further reiterates that the variations in the likelihood of incomplete ANC visits in Nigeria are attributed to the clustering variation in PSUs. The Akaike Information Criterion (AIC) and Schwarz’s Bayesian Information Criterion (BIC) values showed a successive reduction, which means a substantial improvement in each of the models over the previous model, and affirmed the goodness of model III developed in the analysis. Therefore, model III, the complete model with both the selected individual and household-level factors, was selected to predict the likelihood of incomplete ANC visits in Nigeria.

## 4. Discussion

This study examined the spatial pattern and multi-level factors associated with ANC visits in Nigeria using the recent NDHS data collected in 2018. It was observed that areas with high levels of incomplete ANC visits were located in Sokoto, Kebbi, Zamfara, Katsina, Kano, Jigawa, Bauchi, Niger, Borno, Gombe, and Bayelsa. The probable explanation for this finding could be the differences in the level of development across the various regions in the country as well as rural–urban differences, as explained in a comparative study on factors associated with the under-utilization of ANC services in Nigeria by Adewuyi et al. [24], which is also linked to the availability of maternal health services in some of these regions [25,26]. Another possible explanation is the role of sociocultural factors in maternal health services utilization in Nigeria [27,28]. Our results showed statistically significant associations between individual factors, household factors, and ANC visits in Nigeria. At the individual level, the factors associated with incomplete ANC visits included maternal age, maternal education, partner’s level of education, working status, ethnicity, parity, religion, and exposure to media, while the place of residence, wealth index, region, and community literacy level were factors associated with incomplete ANC visits at the household level. On the other hand, marital status, distance to the health facility, sex of household head, and community SES showed no statistically significant associations with incomplete ANC visits.

Among the individual-level factors examined, our findings showed that the likelihood of having an incomplete ANC visit in Nigeria was high among women of reproductive age who had three children and those practicing traditional and other religious beliefs compared to women with one child and those practicing Christianity. This can be explained by the fact that first-time mothers are mostly less experienced and more mindful about the health of their forthcoming baby as compared to multiparous women, hence the differences in ANC visits; this argument is supported by a cross-sectional study on the utilization of antenatal care among rural-to-urban migrant women in Shanghai [29]. Our result is consistent with other studies being carried out across the world, which suggests that parity is inversely related to ANC [29,30,31]. Moreover, completing ANC visits among first-time mothers could be because of time management, more resources in the family, and positive perceptions about the benefits of ANC, which is similar to the findings of Simkhada et al. [32]. Hence, parity influences ANC visits. As parity increases, the experience of timely initiation of ANC decreases as these women might give less value to ANC services than women with one child [30]. Likewise, a woman’s religion was found to be statistically associated with the number of ANC visits. Christian women with one child were less likely to report incomplete ANC visits than women with three children with traditional and other religious backgrounds.

Additionally, our study showed that respondents from Yoruba and Igbo ethnic groups and those exposed to mass media were less likely to report incomplete ANC visits than those from Hausa ethnicity and those not exposed to mass media. This reflects the important role ethnicity, religion, and mass media play in ANC attendance. Comparable to some previous reports, our findings are consistent with the existing literature [24,33,34]. Ethnicity and religion can affect norms and values placed on the use of ANC services.

The contribution of media exposure to ANC visits cannot be underestimated. This was highlighted in our findings. We found a significant positive association between exposure to media and ANC visits. Women exposed to mass media were less likely to report incomplete ANC visits compared to women who were not exposed to mass media. This result is consistent with the findings of Basha [35] and Odesanya et al. [36] in their cross-sectional study on factors affecting the utilization of antenatal care services in Nigeria. They indicated that media access has the power to enlighten women positively about utilizing maternal health care, including ANC services.

Moreover, we found that women of reproductive age who were aged 35 years and above were less likely to report incomplete ANC visits than women of reproductive age between ages 15 and 24. This pattern may be due to the stigmatization of teenage and young mothers. Many teenage and young pregnant women avoid public engagements until after delivery for fear of the social stigma attached to such pregnancy at an immature stage of life. As a result of this stigma, very few ANC contacts are made by such women, and in the extreme, ANC contacts are avoided throughout the pregnancy. This finding is consistent with previous cross-sectional studies in Nigeria by Fagbamigbe et al. and Akanbiemu et al. [33,37].

Again, we found that women with secondary education and above and women whose partners had secondary education and above were less likely to report incomplete ANC visits compared to those with primary and no education. A study in Bangladesh found education to be positively associated with eight or more ANC contacts during pregnancy [38]. Other studies that examined ANC visits in different settings, including Nigeria, have consistently reported that women with higher levels of education have a higher prevalence and greater odds of ANC visits than those with no education [24,39,40]. Thus, maternal education is associated with the use of basic maternal health services, including ANC [40,41], and lack of formal education has been associated with under-utilization or non-use of ANC [24,42]. Maternal education improves health care literacy and makes women appreciate the advantages of using health care services, including ANC (Basha 2019; Mumtaz 2019). In addition, maternal education increases decision-making power and enhances women’s socioeconomic opportunities and status [35,43,44,45] and also their confidence to take action about their health [46,47]. In addition, maternal education and spousal educational attainment were associated with a higher number of ANC visits. This finding emphasizes the importance of education in achieving the recommended ANC visits by the WHO. In Bangladesh, parents’ higher educational attainment was positively associated with having eight or more ANC contacts [38]. A higher level of education among pregnant women and a higher level of education among their spouses may positively influence joint decision-making among couples regarding health-seeking during pregnancy.

At the household/community level, women of reproductive age currently residing in rural areas and those currently residing in the North East were more likely to report incomplete ANC visits than those residing in urban areas and the North Central area. Similar studies on ANC visits in Nigeria have equally reported significant differences in rural–urban utilization of maternal health services, including ANC [35,43,44,45]. The inequities can explain this finding in the distribution of accessible health resources between rural and urban areas [4,24,35,41,48]. In situations where facilities exist in rural areas, they may be inaccessible due to poor road networks, inefficient transport systems, or very far distances, making ANC visits difficult [24,35]. In addition to the aforementioned points, rural areas are inadequately financed. It is difficult to attract and retain health workers in such places, which is consistent with the cross-sectional study of Fagbamigbe and Idemudia in Nigeria [4], making the utilization of maternal health services difficult.

Additionally, lower socioeconomic status and the influence of cultural beliefs and social norms discourage rural women from using ANC compared to their counterparts in urban areas [24,28,49]. These population dynamics could be a plausible explanation for the observed regional differences between women in rural areas and those in the North-Eastern part of Nigeria. Therefore, these findings imply that regional differences in ANC utilization are a possible reflection of educational differences and other community-level factors across the regions.

### 4.1. Strengths and Limitations

This study has various strengths that are worth mentioning. First, we relied on the use of a nationally representative dataset, and hence the findings are applicable to all pregnant women of their reproductive age in Nigeria. Additionally, the use of the Geographical Information System (GIS) in the analysis of the spatial distribution enabled us to identify the hotspots of incomplete ANC visits. However, it is important to interpret the findings against these limitations. The cross-sectional design cannot allow causal interpretation of the findings, but only associations can be made. Recall and social desirability biases cannot be overruled from this study as the number of ANC visits were self-reported. Again, DHS samples are generally not large enough to provide estimates for small geographic areas, which often are needed for monitoring and evaluating decentralized programs. DHS surveys in a specific country are typically conducted every three to six years so that annual estimates of key indicators are not available from the surveys. Finally, the data analysis was restricted to only variables that were in the DHS, and hence some variables might have been excluded.

### 4.2. Policy and Public Health Implications

The findings of this study are relevant to policy and public health practice. The differences in the completion of optimal ANC visits based on spatial analysis emphasize the need to pay attention to the hot spot areas to increase the number of ANC visits for women to benefit from the importance of optimal ANC visits. In addition to this, it is prudent to pay attention to the rural–urban variations and the sociocultural differences associated with ANC uptake. These parameters will need urgent consideration as far as education on the importance of ANC visits by health professionals is concerned. The government could adopt a short-term policy that will make ANC visits compulsory and free for all women within the poor wealth quintile, whilst the long-term policy could be to incentivize ANC visits from eight visits and above.

## 5. Conclusions

It was observed that the hot spot areas were located in Sokoto, Kebbi, Zamfara, Katsina, Kano, Jigawa, Bauchi, Niger, Borno, Gombe, and Bayelsa. The factors associated with incomplete ANC attendance were maternal age, maternal education, partner’s education, working status, ethnicity, parity, religion, exposure to media, place of residence, wealth index, region, and community literacy level. There is a need to consider these factors in designing and strengthening existing interventions aimed at increasing ANC visits to help attain maternal health-related SDGs by 2030.

## Figures and Tables

**Table 1:** Individual- and household-level characteristics of respondents by ANC visits in Nigeria.

Variable (20,003)	Weighted Frequency	Weighted Percentage	Number of ANC Visits		*p*-Value (χ^2^)
Individual Level Factors			Complete	Incomplete	
**Maternal current age**					*p* < 0.001
15–24	4793	23.96	12.59	87.41	
25–34	9665	48.32	22.67	77.33	
35 and above	5545	27.72	22.53	77.47	
**Maternal education**					*p* < 0.001
No education	9245	46.22	4.19	95.81	
Primary education	2913	14.56	19.17	80.83	
Secondary and above	7846	39.22	39.48	60.52	
**Partner’s level of education**					*p* < 0.001
No education	7322	36.60	4.35	95.65	
Primary education	2795	13.97	17.77	82.23	
Secondary and above	9886	49.42	32.66	67.34	
**Marital status**					*p* < 0.001
Currently married	19,387	96.92	19.51	80.49	
Cohabitating	617	3.08	42.25	57.75	
**Working status**					*p* < 0.001
Not working	6393	31.96	10.68	89.32	
Working	13,610	68.04	24.69	75.31	
**Ethnicity**					*p* < 0.001
Hausa	9125	45.62	4.40	95.60	
Yoruba	2428	12.14	61.55	38.45	
Igbo	2424	12.12	44.77	55.23	
Others	6027	30.13	17.63	82.37	
**Parity**					*p* < 0.001
1	3123	15.61	26.36	73.64	
2	3618	18.09	27.47	72.53	
3	3082	15.41	25.39	74.61	
4 and above	10,180	50.89	14.18	85.82	
**Distance to health facility**					*p* < 0.001
Big problem	5685	28.42	15.67	84.33	
Not a big problem	14,319	71.58	22.02	77.98	
**Religion**					
Christianity	7255	36.27	37.93	62.07	
Islam	12,647	63.22	10.16	89.84	
Traditionalist and others	102	0.51	5.83	94.17	
**Exposure to media**					*p* < 0.001
No	7677	38.38	5.60	94.40	
Yes	12,326	61.62	29.31	70.69	
**Household-level factors**					
**Place of residence**					*p* < 0.001
Urban	7737	38.68	35.97	64.03	
Rural	12,266	61.32	10.27	89.73	
**Wealth index**					*p* < 0.001
Poorest	4464	22.32	3.95	96.05	
Poorer	4451	22.25	7.34	92.66	
Middle	4003	20.01	16.85	83.15	
Richer	3671	18.35	31.08	68.92	
Richest	3414	17.07	50.54	49.46	
**Region**					*p* < 0.001
North Central	2806	14.03	14.47	85.53	
North East	3581	17.90	3.78	96.22	
North West	7269	36.34	4.15	95.84	
South East	1874	9.37	38.97	61.03	
South-South	1675	8.38	41.35	58.65	
South West	2798	13.99	63.49	36.51	
**Sex of household head**					
Male	18,595	92.96	19.40	80.60	
Female	1409	7.04	31.00	69.00	
**Community literacy level**					*p* < 0.001
Low	6705	33.52	4.51	95.49	
Medium	6512	32.55	13.97	86.03	
High	6786	33.93	41.73	58.27	
**Community socioeconomic status**					*p* < 0.001
Low	11,957	59.77	8.55	91.45	
Medium	861	4.30	17.10	82.90	
High	7186	35.92	40.00	60.00	

NDHS, 2018.

**Table 2:** SaTScan analysis result summary table for incomplete ANC visits.

Cluster	Enumeration Area (Cluster) Identified	Coordinate/Radius	Population	Case	RR	LLR	*p*-Value
1	336	(12.458115 N, 10.398881 E)/690.22 km	6670	5952	1.79	1307.55	<0.001
2	8	(6.224563 N, 7.971626 E)/16.54 km	109	97	1.28	11.90	0.0062

**Table 3:** Multi-level logistic regression models for individual and household-level factors associated with incomplete ANC visits in Nigeria.

Variable Individual-Level Variables	Model 0	Model I	Model II	Model III
		aOR (95% CI)	aOR (95% CI)	aOR (95% CI)
**Maternal current age**				
15–24		1		1
25–34		0.72 *** [0.62–0.83]		0.82 ** [0.71–0.95]
35 and above		0.58 *** [0.48–0.69]		0.69 *** [0.58–0.83]
**Maternal education**				
No education		1		1
Primary education		0.58 *** [0.48–0.70]		0.73 ** [0.60–0.89]
Secondary and above		0.44 *** [0.36–0.53]		0.62 *** [0.51–0.76]
**Partner’s level of education**				
No education		1		1
Primary education		0.91 [0.74–1.12]		1.04 [0.84–1.29]
Secondary and above		0.63 *** [0.52–0.76]		0.76 ** [0.62–0.93]
**Marital status**				
Currently married		1		1
Cohabitating		0.84 [0.67–1.05]		0.99 [0.79–1.23]
**Working status**				
Not working		1		1
Working		0.75 *** [0.66–0.85]		0.76 *** [0.67–0.87]
**Ethnicity**				
Hausa		1		1
Yoruba		0.12 *** [0.09–0.15]		0.59 ** [0.43–0.80]
Igbo		0.22 *** [0.17–0.29]		0.56 ** [0.40–0.78]
Others		0.56 *** [0.45–0.69]		0.97 [0.76–1.24]
**Parity**				
1		1		1
2		1.14 [0.97–1.33]		1.13 [0.96–1.32]
3		1.28 ** [1.08–1.52]		1.23 * [1.03–1.45]
4 and above		1.65 *** [1.39–1.95]		1.50 *** [1.27–1.77]
**Distance to health facility**				
Big problem		1		1
Not a big problem		0.99 [0.87–1.12]		1.03 [0.91–1.18]
**Religion**				
Christianity		1		1
Islam		1.38 *** [1.17–1.62]		0.98 [0.83–1.16]
Traditionalist and others		5.01 *** [2.12–11.81]		5.79 *** [2.45–13.58]
**Exposure to Media**				
No		1		1
Yes		0.51 *** [0.44–0.59]		0.65 *** [0.56–0.75]
**Household-level factors**				
**Place of residence**				
Urban			1	1
Rural			1.36 ** [1.14–1.64]	1.32 ** [1.10–1.58]
**Wealth index**				
Poorest			1	1
Poorer			0.70 ** [0.56–0.88]	0.80 * [0.63–1.01]
Middle			0.51 *** [0.40–0.65]	0.66 ** [0.52–0.84]
Richer			0.42 *** [0.33–0.54]	0.62 *** [0.47–0.80]
Richest			0.27 *** [0.20–0.35]	0.45 *** [0.33–0.59]
**Region**				
North Central			1	1
North East			4.03 *** [2.97–5.46]	3.00 *** [2.21–4.07]
North West			4.17 *** [3.15–5.52]	3.02 *** [2.19–4.18]
South East			0.37 *** [0.29–0.49]	0.60 ** [0.42–0.84]
South-South			0.45 *** [0.35–0.59]	0.47 *** [0.36–0.61]
South West			0.13 *** [0.10–0.16]	0.19 *** [0.14–0.26]
**Sex of household head**				
Male			1	1
Female			0.95 [0.82–1.11]	1.00 [0.86–1.17]
**Community literacy level**				
Low			1	1
Medium			0.71 * [0.55–0.93]	0.92 [0.70–1.19]
High			0.46 *** [0.34–0.62]	0.72 * [0.53–0.98]
**Community socioeconomic status**				
Low			1	1
Medium			1.06 [0.69–1.64]	1.04 [0.68–1.58]
High			1.11 [0.89–1.38]	1.08 [0.87–1.34]
**Random effects results**				
PSU Variance (95% CI)	5.33 [4.73–6.02]	1.32 [1.13–1.54]	1.17 [1.00–1.37]	1.05 [0.90–1.24]
ICC	0.62	0.29	0.26	0.24
LR Test	χ^2^ = 4953.88, *p* < 0.001	χ^2^ = 840.39, *p* < 0.001	χ^2^ = 769.82, *p* < 0.001	χ^2^ = 636.64, *p* < 0.001
Wald χ^2^	Reference	1420.84 ***	1601.48 ***	1818.00 ***
**Model fitness**				
Log-likelihood	−7161.20	−6617.80	−6499.20	−6379.96
AIC	14,326.39	13,275.60	13,032.40	12,829.91
BIC	14,342.18	13,433.51	13,166.63	13,106.26
Number of clusters	1387	1387	1387	1387

Weighted NDHS, 2018. Exponentiated coefficients; 95% confidence intervals in brackets; aOR = adjusted Odds Ratios; CI = Confidence Interval; 1 = Reference Category; HF: Health facility. * *p* < 0.05; ** *p* < 0.01; *** *p* < 0.001. PSU = Primary Sampling Unit; ICC = Intra-Class Correlation; LR Test = Likelihood Ratio Test; AIC = Akaike’s Information Criterion; BIC = Schwarz’s Bayesian Information Criterion. Model 0 is the null model, a baseline model without any explanatory variables. Model, I is adjusted for individual-level variables (maternal current age, maternal educational level, partner’s educational level, marital status, working status, ethnicity, parity, distance to health facility, religion, and media exposure). Model II is adjusted for household-level variables (place of residence, wealth index, region, sex of household head, community literacy level, and community socioeconomic status). Model III is the final model adjusted for both individual- and household-level variables.

## Data Availability

The datasets utilized in this study can be accessed at https://dhsprogram.com/data/available-datasets.cfm (accessed on 20 March 2021).

## Abbreviations

ANC: Antenatal Care; SSA: sub-Saharan Africa; SDGs: Sustainable Development Goals (SDGs); Nigeria Demographic and Health Survey; NPC: National Population Commission; ICF: International Center for Migration; LLR: Log-Likelihood Ratio, AIC: Akaike Information Criterion; BIC: Schwarz’s Bayesian Information Criterion; VIF: Variance Inflation Factor; RR: Relative Risk; ICC: Intra-Class Correlation; PSUs: Primary Sampling Units; CI: Confidence Interval (CI).
